# Marine Compounds and Autophagy: Beginning of a New Era

**DOI:** 10.3390/md16080260

**Published:** 2018-07-31

**Authors:** Sergey A. Dyshlovoy, Friedemann Honecker

**Affiliations:** 1 Laboratory of Marine Natural Products Chemistry, G.B. Elyakov Pacific Institute of Bioorganic Chemistry, Far-East Branch, Russian Academy of Sciences, 690022 Vladivostok, Russian; 2 Department of Oncology, Hematology and Bone Marrow Transplantation with Section Pneumology, Hubertus Wald-Tumorzentrum, University Medical Center Hamburg-Eppendorf, 20246 Hamburg, Germany; Friedemann.Honecker@zetup.ch; 3 School of Natural Sciences, Far Eastern Federal University, 690022 Vladivostok, Russian; 4 Tumor and Breast Center ZeTuP St. Gallen, CH-9006 St. Gallen, Switzerland

In 2016, the Nobel Prize in Physiology or Medicine was awarded to Prof. Yoshinori Ohsumi for the elucidation of the mechanisms of autophagy. Autophagy (in particular, the so called macroautophagy) is a basic cellular catabolic “self-eating” process which leads to selective or non-selective bulk degradation of organelles and proteins by the lysosome system [1]. Apart from natural stimuli, it can result from cellular stress, e.g., nutrient deprivation or exposure to toxins [2]. It includes the formation of double-membrane vesicles (autophagosomes) that later fuse with lysosomes, leading to the degradation and recycling of sequestered contents [1]. This cellular process is implicated in many aspects of cell physiology and plays a particular role in the survival and death of mammalian cells. It is highly important in maintaining tissue homeostasis, and is involved in the pathophysiology of human diseases ranging from cancer to neurodegenerative conditions (Parkinson’s and Alzheimer’s disease), cardiomyopathy, and others. From a therapeutic point of view, it is an exciting perspective to find and develop compounds that have the ability to control and modify this process. In the cellular context, the biological effect of autophagy can be either cytotoxic (programmed cell death type II), cytoprotective, cytostatic, or even non-protective (meaning that it does not affect cell viability or the sensitivity of cells to certain drugs) [3]. At present, autophagy has been much less studied in comparison with other basic biological processes like apoptosis (programmed cell death type I). However, in recent years, the critical role of autophagy in a number of physiological events has become clearer, which has gained a lot of attention for the field.

Lysosomes, which are an essential part of the autophagy machinery, play a role at the final stages of this process, providing the degradation of the content of autophagosomes. Furthermore, lysosomes participate in a vast number of physiological and pathological processes, and evidence is accumulating that they play a role in both drug sensitivity and drug resistance. For instance, it is known that several clinically approved compounds target lysosomes as their mode of action. This has sparked the interest of research groups to identify new substances that are able to modulate autophagy via targeting of lysosome and/or other organelles and molecules.

Compared to terrestrial life forms, marine inhabitants are by far less well studied organisms. At the same time, due to the extreme environmental conditions (high pressure, lack of light, salinity, pH), they harbor a unique variety of chemical compounds, of which a large number still await discovery and characterization. A good proportion of these compounds exhibit potent biological activity, targeting one or several specific biological processes. In recent years, several new and previously known marine-derived compounds have been found to exhibit autophagy-modulatory activity sometimes determining their biological activity, e.g., anticancer effects. Due to the often limited access to marine-derived substances on the one hand, and the relative novelty of autophagy as a topic in cell biology, studies of marine compounds targeting this promising process are still rather limited. However, we expect that the field will show exponential growth within the next few years.

Our Special Issue “Marine Compounds as Modulators of Autophagy and Lysosomal Activity” (http://www.mdpi.com/journal/marinedrugs/special_issues/marine_autophagy) as part of the open access journal *Marine Drugs* (ISSN 1660-3397) started in 2016. In total, four high-quality papers have been published in the Special Issue including one review and three original articles. The Special Issue covered both novel chemical and biological aspects of autophagy and lysosome-related analyses.

Since autophagy is a relatively new and sometimes still controversial topic, results of similar experiments have often been interpreted in different ways. Therefore, we have suggested to the authors publishing in the current Special Issue to use the recommendations recently described by Klionsky et al. [4] for the interpretation of experimental data.

Ruocco and colleagues from Napoli (Italy) provided a thorough review entitled “**Blue-Print Autophagy: Potential for Cancer Treatment**” [5]. In this manuscript, the authors provided a general overview on marine natural products involved in the modulation (both stimulation and inhibition) of autophagy with respect to their potential as cancer treatments. The authors concluded that so called blue-print autophagy provides a new avenue for cancer treatment [5]. Ratovitski from Baltimore (USA) reported on three marine-derived compounds, namely **chromomycin A2**, **psammaplin A**, and **ilimaquinone**, which can induce the expression of several autophagic signaling intermediates in three types of human cancer cells in vitro (human squamous cell carcinoma, glioblastoma, and colorectal carcinoma) [6]. This effect appears to be transcriptionally regulated by TP53 family members. This research has revealed some novel aspects of the mechanisms of action of these previously known marine compounds [6]. Using a proteomics approach, Li and colleagues from Guangdong (China) examined the effect and mechanism of action of **tachyplesin I** (cationic peptide from the horseshoe crab *Tachypleus tridentatus*) on human glioblastoma cells using a three-dimensional neurospheroid model [7]. Among other effects, they found that tachyplesin I affects lysosomes, in particular reducing the expression of several lysosomal hydrolases in this cell model. Additionally, this peptide alters cellular glycolysis and up-regulates topoisomerase 2-alpha, contributing to its anti-tumor effect [7]. Finally, Wan and colleagues from Corvallis (USA) and Dundee (UK) investigated the autophagy-inducing mechanism of the marine compounds **coibamide A** and **apratoxin A** [8]. They found that ATG5 was required for coibamide-induced mTOR-independent autophagy, but was dispensable for coibamide-induced apoptosis. Both compounds promoted cross-signaling between ATG5-dependent autophagy and caspase-dependent apoptosis, and may be valuable tools for the analysis of alternative modes of regulated cell death in mammalian cells [8].

In summary, our Special Issue “Marine Compounds as Modulators of Autophagy and Lysosomal Activity” in *Marine Drugs* compiles the results of research activities from the field of autophagy and marine compounds from the years 2016 and 2017. We are grateful to all authors who contributed to our Special Issue, and we are looking forward to new and exciting discoveries!
